# Supplementary figures and images for: The impact of delayed evacuation on the quality of human fetal tissue
Source: PLoS One. 2026 Jan 21;21(1):e0328595. doi: 10.1371/journal.pone.0328595 (PMC12822946; doi:10.1371/journal.pone.0328595)

**S1 Fig. DNA Quality with Delayed Evacuation Compared to Immediate Evacuation.**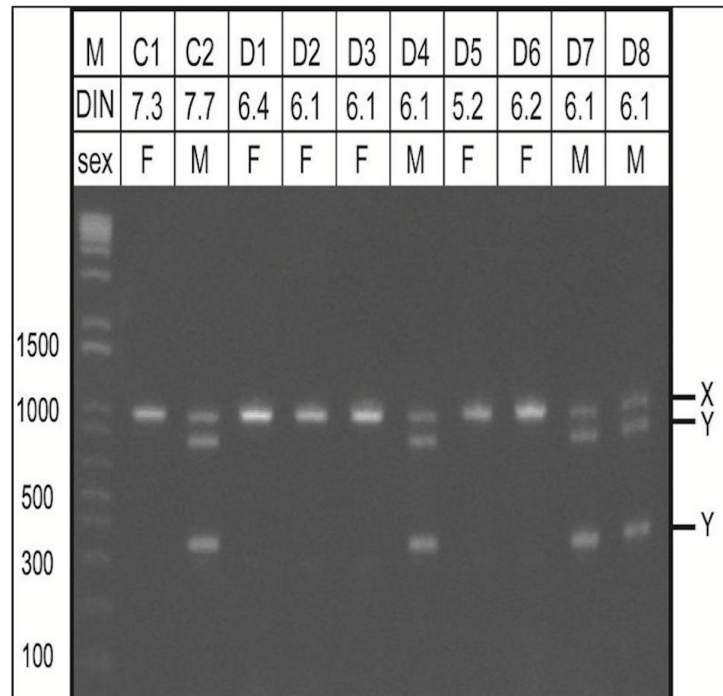

Supplement: S1 Fig — Sex-specific PCR products. Two IE specimens (C1-C2) and eight DE specimens (D1-D8) visualized on a 1% agarose E-Gel (Life Technologies). DNA integrity numbers (DIN) are shown above the gel. Specimens C2, D4, and D7-8 all had male external genitalia and amplified both X (977 base-pairs) and Y (790 and 358 base-pairs) chromosome-specific PCR products, while C1, D1-3, and D5-6 had female external genitalia and amplified only the X chromosome-specific PCR product. The DNA ladder (M) used is E-Gel 1Kb Plus DNA Ladder (Life Technologies #10488−090). (PDF) [file pone.0328595.s001.pdf]
