# Supplementary material for: The impact of delayed evacuation on the quality of human fetal tissue
Source: PLoS One. 2026 Jan 21;21(1):e0328595. doi: 10.1371/journal.pone.0328595 (PMC12822946; doi:10.1371/journal.pone.0328595)
Supplement: S2 Table — Symbols indicate the proportion of cells with stain uptake compared to control tissue: (+++) 76–100%, (++) 50–75%, (+) 25–49%, (-) 0–24%. Semi-quantitative assessments of apoptosis (TUNEL) and proliferation (Ki67) in tissues from three DE specimens evacuated 19–25 hours after digoxin injection compared to tissues from IE specimens. Semi-quantitative assessments of apoptosis (TUNEL) and proliferation (Ki67) in tissues from two IE specimens incubated at 37°C for 0, 6, and 18 hours. (PDF) [file pone.0328595.s003.pdf]

S1 Fig. DNA Quality with Delayed Evacuation Compared to Immediate Evacuation.

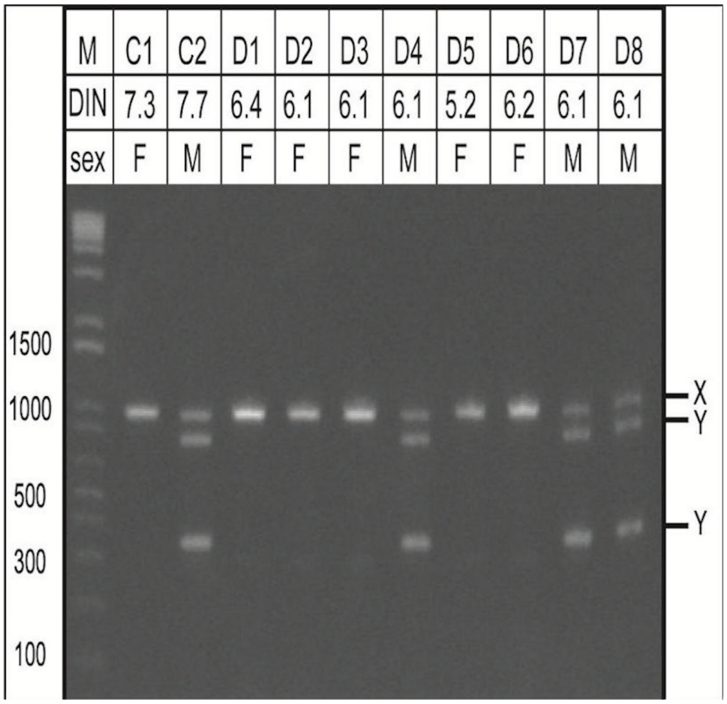

S2 Table. Tissue Quality Assessment by Histochemistry (TUNEL) and Immunohistochemistry (Ki67).

	Additional time at 37°C		0 hours		6 hours		18 hours	
			TUNEL	Ki67	TUNEL	Ki67	TUNEL	Ki67
Delayed evacuation	Specimen #15	Brain	+++	+				
		Kidney	+++	-				
		Lung	+++	+				
		Muscle	+	+				
	Specimen #16	Brain	+	+				
		Kidney	+++	-				
		Lung	+,++	-				
		Muscle	-	+,++				
	Specimen #17	Brain	+++	++				
		Kidney	+++	-				
		Lung	+++	-				
		Muscle	+	+				
Immediate evacuation	Specimen #40	Brain	-	+++	-	+,++	+++	-
		Kidney	-	+++	++	++	+++	-
		Lung	-	+++	+	+	++,+++	-
		Muscle	-	+++	-	++	++	-
	Specimen #32	Brain	-	+++		+,++	+++	-
		Kidney	-	+++	++	++	+++	-
		Lung	-	+++	+	+	+++	-
		Muscle	-	+++	+	++	+++	-
